# Supplementary material for: Genome-Wide Analysis of the WRKY Gene Family in Malus domestica and the Role of MdWRKY70L in Response to Drought and Salt Stresses
Source: Genes (Basel). 2022 Jun 15;13(6):1068. doi: 10.3390/genes13061068 (PMC9222762; doi:10.3390/genes13061068)
Supplement: Supplementary file 1 [file genes-13-01068-s001.zip › genes-1760034-supplementary.pdf]

Supplementary Table S1. Primers information and sequences in this study.

Name	Purpose/Vector	Primer sequences (5′-3′)
MD03G1057400	Clone	F: ATGGCCTCCTCTTCAGGG<br>R: ATGGCCTCCTCTTCAGGG
MD17G1223100		F:ATGGACCATTCAGCTGCATATGATG<br>R:CAGTCTAACAAGTGAAACACCAATG
MD08G1127200		F: ATGACGGTGGAGCTGATGGAAT<br>R: TCACGTTGACTCGTACACCAAC
MD09G1150700		F: CTCATCTTCTTCTACCATGTCAAAT<br>R: GCCCGCCAATCGACTCGAAAA
MD00G1140800		F: ATGGATGAAACCCTTTCTCCAGAAC<br>R:TCAAGAAGGGACAGAGGGAATCCTT
MD15G1106600		F: ATGGCGGTGGAGCTGATGAACTTC<br>R: TCACGTCGACTCAGACACCAAC
MD01G1168600		F:TCAAGAAGGGACAGAGGGAATCCTT<br>R:CATCACTGCGAGGAGTTCATAGGAA
MD03G1057400	2300-EGFP	F: CGAGCTCGGTACCCGGGGATCCATGGCCTCCTCTTCAGGGAG<br>R: CCTTGCTCACCATGGTGTCGACGCACAGCAATGATTCAAAAA
MD17G1223100		F: CGAGCTCGGTACCCGGGGATCCATGGACCATTCAGCTGCATA<br>R: CGAGCTCGGTACCCGGGGATCCATGGACCATTCAGCTGCATA
MD08G1127200		F:CGAGCTCGGTACCCGGGGATCCATGACGGTGGAGCTGATGGA<br>R:CGAGCTCGGTACCCGGGGATCCATGACGGTGGAGCTGATGGA
MD09G1150700		F:CGAGCTCGGTACCCGGGGATCCATGTCAAATGAGAAGAAAAA<br>R:CCTTGCTCACCATGGTGTCGACTGGCTCTTTTTTGTGACTAA
MD00G1140800		F: CCTTGCTCACCATGGTGTCGACTGGCTCTTTTTTGTGACTAA<br>R:CCTTGCTCACCATGGTGTCGACAGAAGGGACAGAGGGAATCC
MD15G1106600		F:CGAGCTCGGTACCCGGGGATCCATGGCGGTGGAGCTGATGAA<br>R: CCTTGCTCACCATGGTGTCGACCGTCGACTCAGACACCAACC
MD01G1168600		F: CCTTGCTCACCATGGTGTCGACAGAAGGGACAGAGGGAATCC<br>R: CCTTGCTCACCATGGTGTCGACTAGGAAACTCTCAAAATTAA
MD01G1168600	qRT-PCR	F: TGCTGTCGGAGATGAGATAA<br>R: TTTGGTGCTTTCAGATCCCT
MDH		F: CGTGATTGGGTACTTGGAAC<br>R: TGGCAAGTGACTGGGAATGA

Supplementary Table S2. Synteny relationship of *WRKY* genes between *Arabidopsis* and *Malus domestica*.

Pairs 1		Pairs 2		Pairs 3	
AtWRKYs	MdWRKYs	AtWRKYs	MdWRKYs	AtWRKYs	MdWRKYs
AT5G15130	MD17G1048400	AT1G80840	MD09G1224500	AT3G04670	MD04G1226400
AT4G39410	MD15G1337100	AT3G01970	MD09G1008800	AT5G28650	
AT4G31550	MD15G1106600	AT5G13080		AT2G37260	MD04G1113100
AT4G31800	MD15G1039500	AT2G24570	MD08G1127200	AT4G26640	MD03G1188900
AT2G25000		AT5G24110	MD07G1285200	AT2G23320	MD02G1177500
AT5G49520	MD13G1150700	AT2G44745	MD07G1110400	AT4G30935	MD02G1007900
AT2G03340	MD13G1067600	AT4G11070	MD06G1104100	AT5G52830	MD01G1210200
AT1G13960		AT4G23810		AT2G40750	MD01G1168600
AT1G69310	MD13G1064700	AT4G22070	MD05G1349800	AT3G56400	
AT2G38470	MD12G1181000	AT4G04450		AT2G40740	MD01G1168500
AT2G30250	MD11G1059400	AT1G62300		AT2G46400	MD01G1078000
AT4G18170	MD10G1266400	AT1G29280	MD05G1295700	AT2G46130	MD01G1071600
AT2G47260	MD09G1285400	AT1G29860	MD05G1290300	AT4G01250	MD01G1071300

Note: the synteny relationship existed between AtWRKYs and MdWRKYs in Pairs 1, 2 and 3.

Supplementary Table S3. Conserved motifs found in the MdWRKY proteins via MEME.

Motif	Width	E-value	Logo
1	23	4.7e-1680	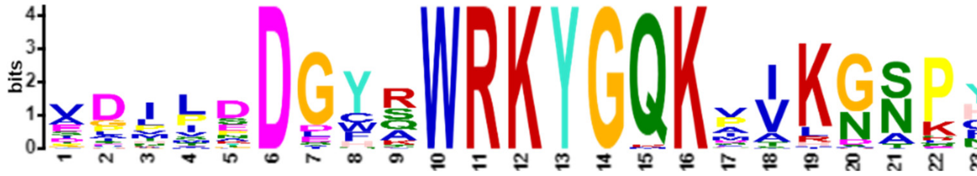
2	29	3.0e-2049	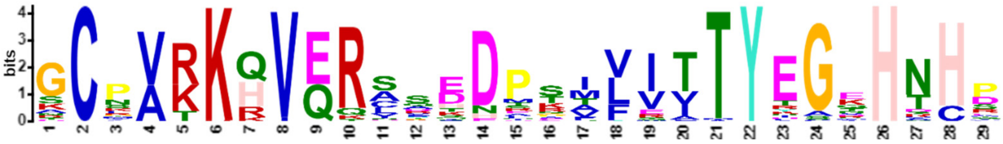
3	8	3.4e-695	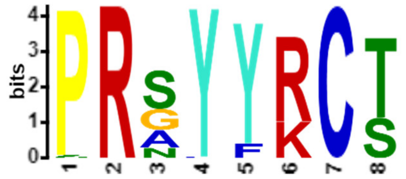
4	31	8.7e-423	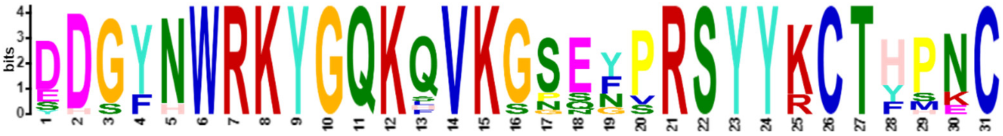
5	15	6.0e-244	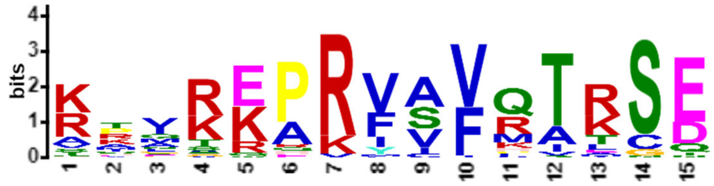
6	15	6.9e-154	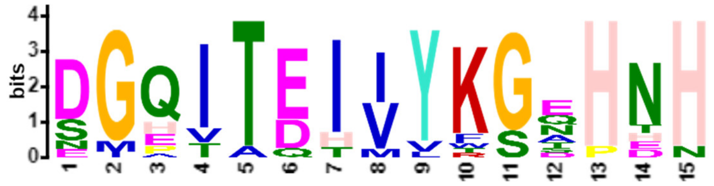

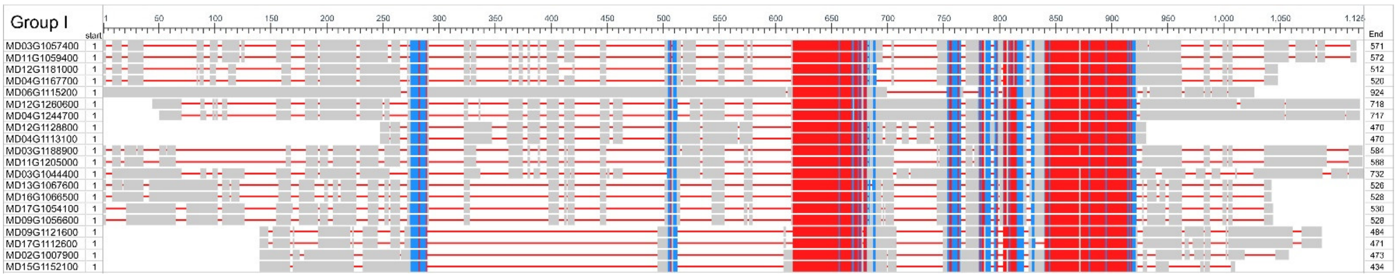

Supplementary Figure S1. Multiple alignment of MdWRKYs in group I. Only the highly conserved and less conserved amino acid positions were highlighted based on the relative entropy threshold of the residue. Only alignment positions with no gaps will be colored. Red box indicated highly conserved positions and blue box indicated lower conservation, grey box indicated nonconserved positions, and red line indicated gaps.

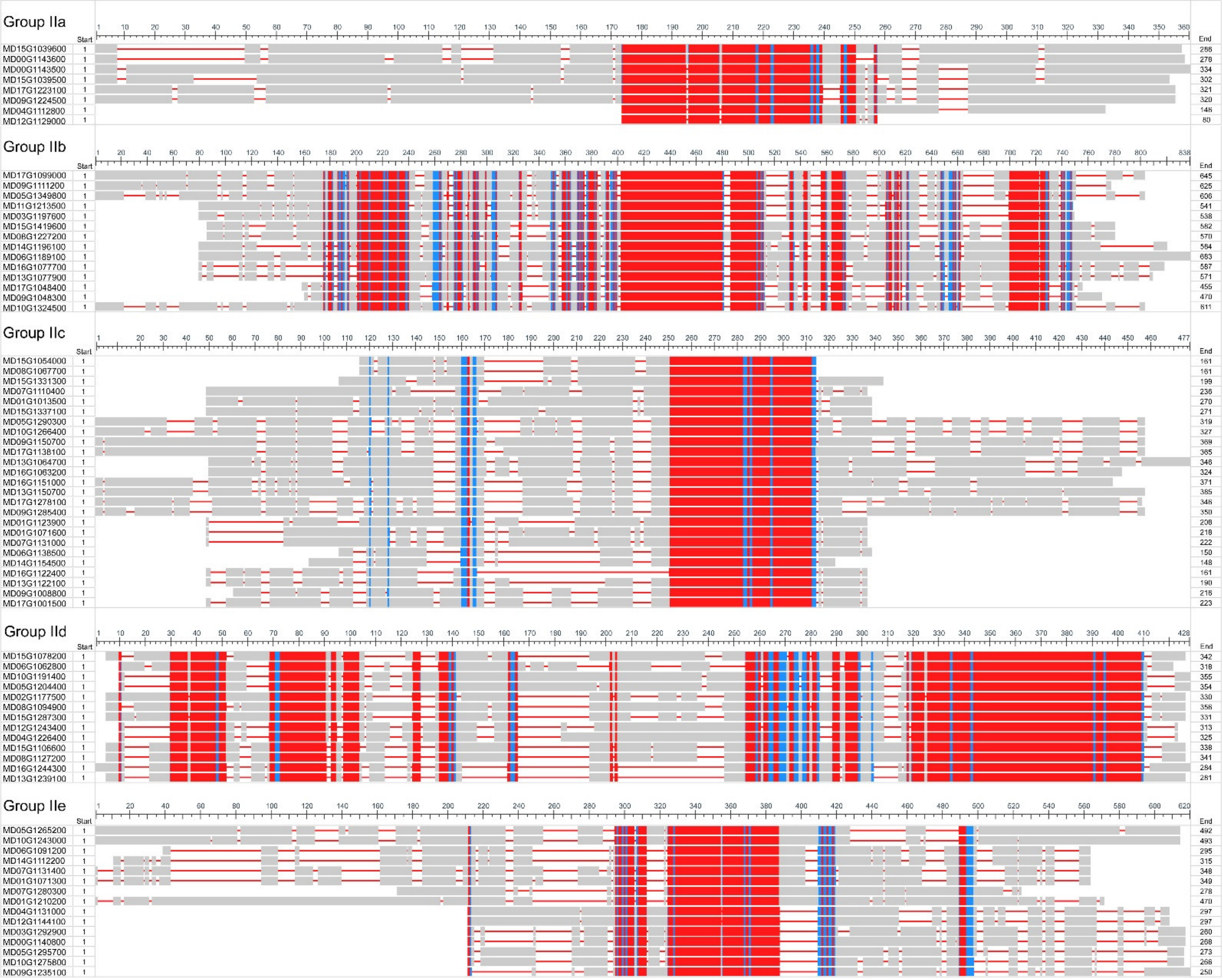

Supplementary Figure S2. Multiple alignment of MdWRKYs in group II. Only the highly conserved and less conserved amino acid positions were highlighted based on the relative entropy threshold of the residue. Only alignment positions with no gaps will be colored. Red box indicated highly conserved positions and blue box indicated lower conservation, grey box indicated nonconserved positions, and red line indicated gaps.

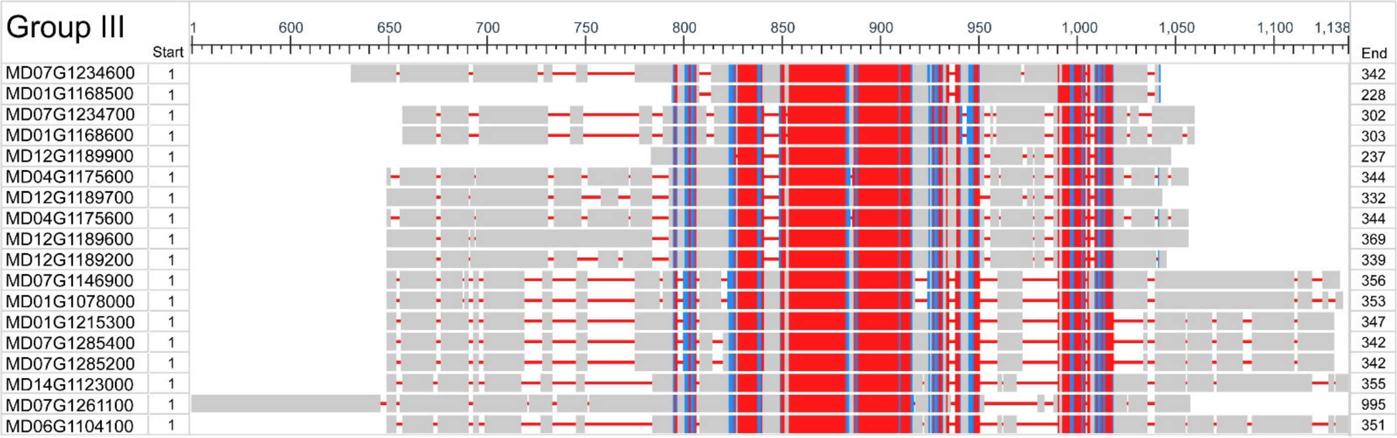

Supplementary Figure S3: Multiple alignment of MdWRKYs in group III. Only the highly conserved and less conserved amino acid positions were highlighted based on the relative entropy threshold of the residue. Only alignment positions with no gaps will be colored. Red box indicated highly conserved positions and blue box indicated lower conservation, grey box indicated nonconserved positions, and red line indicated gaps.

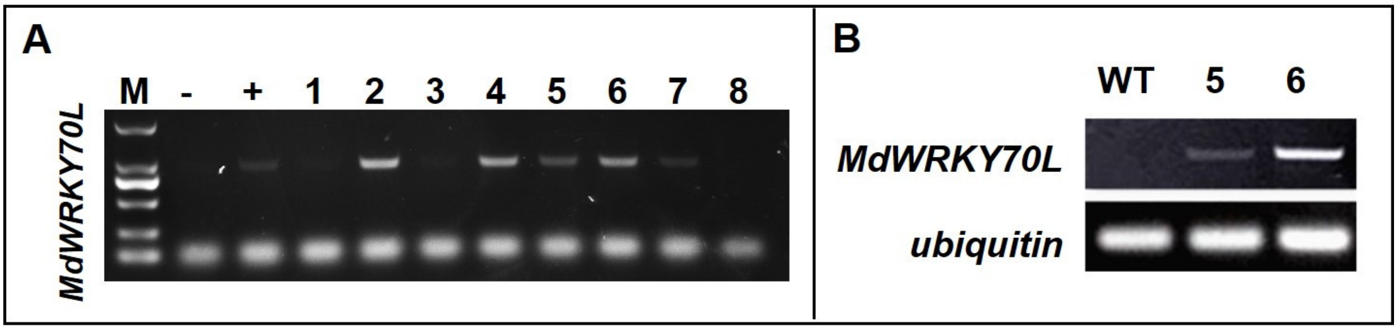

Supplementary Figure S4. Identification of transgenic tobacco plants. (**A**) PCR analysis of the regenerated tobacco by gene specific primers. (**B**) Expression analysis of *MdWRKY70L* in the transgenic tobacco and wild type (WT).
